# *Strychni Semen* Improves Spatial Memory and Promotes Axon Regeneration Following Vascular Dementia in Mice

**DOI:** 10.2174/011570159X388589251030173848

**Published:** 2026-01-07

**Authors:** Yan Zhang, Mengting Liu, Xinyue Zhao, Shuhui Deng, Zhuchen Zhou, Bingjie Wan, Yuting Yan, Xuhong Jiang, Zhong Chen, Yanrong Zheng

**Affiliations:** 1 Zhejiang Key Laboratory of Neuropsychopharmacology, School of Pharmaceutical Sciences, Jinhua Academy, Zhejiang Chinese Medical University, Hangzhou, Zhejiang, 310053, China;; 2 First Affiliated Hospital of Zhejiang Chinese Medical University, Hangzhou, Zhejiang, 310006, China

**Keywords:** Vascular dementia, axon regeneration, mitochondrial transport, Strychni Semen, brucine, strychnine

## Abstract

**Introduction:**

Vascular dementia (VaD) is a cognitive disorder caused by cerebrovascular lesions, often accompanied by secondary axonal damage. Given the high energy-consuming nature of neurons, efficient mitochondrial transport is essential for providing sufficient energy for axonal regrowth. However, developing drugs that effectively modulate mitochondrial transport and support axonal regeneration following VaD remains a challenge.

**Methods:**

Bilateral carotid artery stenosis (BCAS) was induced in mice, and cognitive function was assessed using the Morris water maze test. Axonal regeneration was quantified *via * anterograde viral tracing and immunofluorescence. The oxygen-glucose deprivation (OGD) model was established in primary cortical neurons. Axonal elongation and mitochondrial motility were evaluated using live-cell imaging. Western blotting and molecular docking techniques were applied to decipher the molecular mechanisms.

**Results:**

Our study found that * Strychni Semen* and its active compound, strychnine (but not brucine), improved cognitive function and promoted axonal regeneration in mice subjected to BCAS. Strychnine promotes post-OGD axonal mitochondrial transport, and inhibiting mitochondrial transport blocks its axon regeneration-promoting effects. Moreover, strychnine augmented the translocation of several transporter proteins to mitochondria. Molecular docking studies further indicate that the proteins DISC1 and FEZ1 may play pivotal roles in strychnine-induced mitochondrial transport.

**Discussion:**

This study provides preliminary evidence supporting the therapeutic potential of *Strychni Semen* and its active compound, strychnine, in axonal regeneration and VaD, although the underlying molecular machinery requires further elucidation.

**Conclusion:**

Our results indicate that *Strychni Semen* and its active compound, strychnine, encourage axonal regeneration following VaD in mice by enhancing axonal mitochondrial transport.

## 1 INTRODUCTION

Vascular dementia (VaD) is the second most prevalent type of dementia after Alzheimer’s disease. The clinical manifestation of VaD shows cognitive decline resulting from cerebrovascular pathology [1]. The increasing incidence of VaD presents significant social and economic challenges for patients and their families [2]. The pathological process of VaD is complex and multifaceted, including neuronal damage, axonal degeneration, and synaptic dysfunction [3, 4]. Despite advancements in understanding VaD mechanisms [5], therapeutic options remain limited [6]. Axons are the elongated projections of neurons that facilitate signal transmission, are particularly vulnerable in VaD. Hypoxic conditions due to cerebrovascular damage disrupt axonal integrity, induce mitochondrial dysfunction in axons, and disrupt neural signaling [3, 4]. Axonal lesions structurally exhibit swelling, discontinuity, and demyelination [7-9], while functionally impairing the neuron’s ability to conduct electrical impulses [10, 11]. Therefore, promoting axonal regeneration has been considered a potential strategy for improving cognitive function in VaD patients. However, effective strategies for stimulating central axonal regrowth are currently lacking.

Mitochondria act as the powerhouses of the cell [12], playing a crucial role in axonal regeneration [13]. Beyond supplying essential energy for axonal repair [14], mitochondria regulate intracellular calcium levels and facilitate signaling processes involved in both degeneration and regeneration [15-17]. Recent research also highlights the significance of intracellular mitochondrial transport in axonal regeneration [18]. In the peripheral spinal cord and sciatic nerve injuries, genetic deletion of syntaphilin (SNPH) can enhance mitochondrial mobility, thereby promoting axonal regeneration post-injury [19, 20]. Similarly, SNPH deletion following axotomy has been shown to increase mitochondrial transport and support axonal regeneration in cortical neurons in the central nervous system (CNS) [20]. Enhancement of mitochondrial transport occurs along axons at the injured part to ensure a greater presence of healthy mitochondria, supplying the necessary energy for axonal regrowth [20-22]. However, neuronal injuries often lead to impaired mitochondrial function and transport, which then hinder the regeneration process. Current methods to promote mitochondrial transport in axons mainly involve genetic manipulation, while pharmacological approaches are hardly available. Therefore, the development of drugs to effectively modulate mitochondrial transport and support axonal regeneration remains an urgent challenge.

Traditional Chinese medicine (TCM) has demonstrated significant potential in the treatment of neurological diseases, such as ischemia, Alzheimer’s disease, and Parkinson’s disease [23-26]. *Strychni Semen* is the dried mature seeds of *Strychnos nux-vomica* L., and its active compounds (brucine and strychnine) [27] have been demonstrated to be neuroprotective [28-30]. Additionally, substantial evidence supports their applications in pain relief and anti-inflammatory treatments [27]. Notably, *Strychni Semen* and its active compounds can treat stroke [31]. In the middle cerebral artery occlusion (MCAO) model, *Strychni Semen* treatment promoted neurological recovery after the MCAO model in rats [32]. However, the underlying mechanisms remain unclear, and the role of *Strychni Semen* and its active compounds in promoting axonal regeneration, particularly in VaD, remains to be explored.

Based on the background, our study aims to investigate the impact of *Strychni Semen* and its active compounds, strychnine and brucine, on axonal regeneration following VaD, and to explore the underlying mechanisms. We established a mouse model of VaD (bilateral carotid artery stenosis, BCAS) to evaluate the effects of *Strychni Semen* and its active compounds on cognitive function [33, 34], and further analyzed their roles in axonal regeneration. Furthermore, we combined histological analysis and molecular biology techniques to investigate their impact on axonal mitochondrial transport. In summary, this study not only provides new insights into the role of *Strychni Semen* in neural repair but also contributes to the development of novel therapeutic strategies and targets for VaD.

## 2 MATERIALS AND METHODS

### 2.1 Reagents

*Strychni Semen* is purchased from the First Affiliated Hospital of Zhejiang Chinese Medical University (Zhejiang Provincial Hospital of Traditional Chinese Medicine) (Chinese medicine preparation approval, Z20100263) [35]. *Strychni Semen* (origin: Yunnan, China) was prepared as previously reported [35]. Briefly, the raw seeds were cleaned and soaked in water for 10 days (changing the water every 3 days in winter, daily in summer) until they were fully hydrated and devoid of any hard core inside. Subsequently, the hydrated seeds were de-skinned, thinly sliced, and thoroughly dried. The dried slices were then fried in sesame oil until they turned brownish-yellow. Talcum powder was added to absorb excess sesame oil. The slices were rinsed thoroughly with clean water to remove the talcum powder and subsequently dried in the sun for storage. Finally, the processed slices were ground into a fine powder using a 100-mesh sieve. The dosage of *Strychni Semen* based on body surface area normalization, the human clinical dose of 1.2 g per day was converted to an equivalent mouse dose of ~150 mg/kg (1200 mg/70 kg × 9.1) for preclinical studies [35]. This dose is well below the LD_50_ of 564.55 mg/kg, ensuring experimental safety. Brucine was purchased from Ron Reagent Company (China) with a content of ≥ 98%, and Strychnine was purchased from Aladdin Reagent Company (China) with a content of ≥ 99%. The doses of brucine and strychnine were determined based on previous studies [36], all of which fell within the established safety ranges for mice. All medications are dissolved in physiological saline.

### 2.2 Animal

8-week-old C57BL/6J wild-type male mice (20-25 g) and pregnant 10-week-old female mice (gestational days 14-16, 30-40 g) were purchased from Hangzhou Qizhen Laboratory Animal Co., Ltd. The animals were housed in a room with controlled humidity (55 ± 10%), a stable temperature (22-24°C), and a 12-hour light-dark cycle (light time: 8:30-20:30). Mice were provided with food and water ad libitum. All mice were maintained under these conditions for 2 weeks before the experiment. Based on a block randomization strategy (random numbers were generated using the RAND function in Microsoft Excel), all animals were randomly divided into control, BCAS, and treatment groups. The health status of the animals was monitored daily through evaluations of body weight, activity, breathing patterns, and fur condition. Mice were subjected to euthanasia if they exhibited a rapid loss of 20% of their initial body weight. Efforts were made to minimize the number of animals used and to reduce animal suffering during the experiments. The number of animals used in each experiment has been specified in the figure legends. The use and care of laboratory mice strictly complied with the ethical guidelines of the Animal Advisory Committee of Zhejiang Chinese Medical University and the US National Institutes of Health Guidelines for the Care and Use of Laboratory Animals. All procedures were approved by the Animal Advisory Committee of Zhejiang Chinese Medical University (No. 15399).

### 2.3 Surgical Procedures

 To induce BCAS, mice were anesthetized with sodium pentobarbital (100 mg/kg, intraperitoneally). The mice were placed supine on a heating pad, and a midline incision was made along the neck to expose the common carotid artery. The nerve was carefully separated, and a microcoil with a diameter of 0.18 mm (Beijing Cinontech Co., Ltd., China) was introduced around one side of the artery. The wound was kept moist with a sterile cotton ball saturated with saline solution. After 30 minutes, the procedure was repeated on the contralateral side. The tissue and skin were carefully arranged and sutured with 6-0 sutures upon completion. Animals were returned to the normal cage until they returned to normal activity from anesthesia with free access to food and water [33, 34].

### 2.4 Viral Injections

Sodium pentobarbital (100 mg/kg intraperitoneally) was administered to anesthetize the mice, and placed in a stereotaxic frame (RWD Life Science, China). During the total operation, the body temperature of the anesthetized mice was constantly maintained at 37°C using a heating pad. After shaving the hair, the scalp was disinfected with iodine solution. The skin on the mice’s head was cut open with scissors, and the skull was cleaned with a cotton swab to expose the skull surface. The viruses were injected unilaterally at the coordinates: from bregma, anteroposterior (AP): 0.0 mm; (mediolateral) ML: -3.0 mm, dorsoventral (DV): -2.0 mm. The pre-pulled glass electrode was fixed to the tip of a 1 μL microsyringe using hot melt adhesive, and 100 nL of viral solution (AAV-CMV-EGFP) was injected at a rate of 20 nL/min using a microinjection pump (Micro 4, World Precision Instruments, USA). After viral infusion, the needle was kept at the injection site for 5 min and then slowly withdrawn to reduce backflow. The mice’s skin was sutured using sutures or skin staples. After the surgery, the animals were placed in a warming box until they recovered from anesthesia and resumed normal activity, after which it was returned to their housing cage. The sample size in this experiment (n ≥ 3) was chosen based on previous studies [37].

### 2.5 Primary Cortical Neurons Culture and OGD Procedures

For the culture of primary cortical neurons, pregnant mice with embryonic fetuses (embryonic day 14-16) were used. Pregnant mice were sacrificed and dissected after ether anesthesia. Fetal mice were collected into pre-chilled Hank’s balanced salt solution (HBSS) (14175095, Gibco, USA) on ice. Carefully isolate cortical tissues from brains. The cortex was transferred to a new petri dish containing HBSS and kept on ice throughout the process. The vascular membrane was removed from the isolated tissue, which was then minced and digested with 0.25% trypsin (25200056, Gibco, USA) in a 37°C water bath for 30 minutes. Digestion was terminated by adding Dulbecco’s modified Eagle medium (DMEM) (11965092, Gibco, USA) medium with 10% fetal bovine serum (FBS) (16000044, Gibco, USA). The brain tissue was dissociated into a single-cell suspension by repeated aspiration using a Pasteur pipette. Cells were seeded in 10 cm petri dishes or microfluidic devices (XC450, Xona, USA) coated with poly-L-lysine (P1399, Sigma, USA) and cultured at 37°C in a 5% CO_2_ incubator. Primary cortical neurons were cultured *in vitro* for 7 days before treatment.

For oxygen-glucose deprivation (OGD) treatment, neuronal cells cultured for 7 days at 37°C and 5% CO_2_ were rinsed with glucose-free DMEM (11966025, Gibco, USA). The cells were then placed in a hypoxic chamber (Billups-Rothenberg, MIC-101, USA) containing 5% CO_2_ and 95% N_2_ and incubated at 37°C for 2 hours. For reperfusion, the cells were returned to their usual neuronal culture medium. Subsequent procedures were carried out after 1 or 3 days. Our previous study [22] demonstrated that mitochondrial motility begins to recover 24 hours post-reoxygenation. However, axonal regrowth, a structurally and functionally complex process, requires a prolonged time to manifest visibly. Therefore, we chose 3 days as the detection time point. Cells were included in the regrowth measurement study if they successfully underwent OGD and maintained growth (confluency > 50%) for 3 days. Cell groups that failed to meet the experimental requirements were excluded.

### 2.6 Morris Water Maze

The following behavioral tests were performed on mice that had undergone BCAS or sham surgery after 21 days, as part of the neurological recovery assessment.

Spatial learning and memory abilities of mice were assessed using the Morris water maze (MWM) as previously described [38, 39]. Testing sessions were scheduled between 8:30 and 20:30, with the order of tests randomized each day. Each animal was tested at varying times across different testing days. The experimental setup consisted of a circular pool with a diameter of 120 cm, divided into four equal quadrants. A circular platform with a diameter of approximately 6 cm was placed in the third quadrant, and visual cues for spatial orientation were set up in each quadrant. Data were recorded and analyzed using Anymaze software. On the first day of the experiment, the platform was made visible, protruding about 1 cm above the water surface to allow the mice to see and find it easily. From days 2 to 5, during the hidden platform training phase, the platform was submerged about 1 cm below the water surface, making it invisible to the mice. Mice were placed in the water from four different entry points and allowed to swim freely to locate the platform within 60 seconds. If a mouse successfully reached the platform within 60 seconds, the time taken was recorded. If the mouse did not locate the platform within this time, it was guided to the platform and kept there for 10 seconds. Following the hidden platform training phase, the no-platform trial was conducted. The platform was removed, and animals were placed into the pool from an entry point in quadrant 1, allowing them to swim and explore freely. The swimming trajectory and the number of times the mouse crossed the previous platform location within 60 seconds were recorded. Spatial learning and memory abilities were assessed by comparing the time taken for the mice to reach the target platform location and the number of times they crossed the platform area. Animals were included in the study if they completed the MWM test successfully. Animals that failed to reach the target platform in MWM learning or failed to complete the test were excluded. The sample size in this experiment (n = 10) was chosen based on previous studies [40].

### 2.7 Inhibition of Mitochondrial Motility and Real-time Axonal Mitochondrial Motion Imaging Analysis

The inhibition of mitochondrial motility was performed after 7 days of *in vitro* culture of primary neurons. Mitochondrial motility was inhibited using a mitochondrial Miro1 reducer (HY-163172, Sigma, USA) at a concentration of 10 μM for 6 h.

 To monitor axonal mitochondrial motility, neurons were cultured in a microfluidic control chamber. On the day of imaging, the culture medium was replaced with neuronal culture medium containing Mitotracker (50 nM, Cat. No. 40742ES50, Yeasen, China) and incubated for 20 minutes. The medium was then refreshed with new neuronal culture medium. Images were acquired every 10 seconds at a resolution of 1024 × 1024 pixels for 10 minutes using a confocal microscope at 10X magnification. Mitochondrial motility in axons was quantified using methods previously described [22]. Axons were straightened by Image J with the ‘Straighten’ tool, and kymographs were generated with the ‘Kymograph’ tool. Mitochondria with movement distances less than 5μm were considered stationary, whereas those exceeding 5μm were classified as motile. The velocity of each mitochondrion was calculated based on the slope of the lines in the kymograph. The sample size in this experiment (n = 40 axons from 4 independent experiments for each group) was chosen based on previous studies [41, 42].

### 2.8 Immunofluorescence Staining

Mice were deeply anesthetized with sodium pentobarbital (100 mg/kg intraperitoneally). Phosphate-buffered saline (PBS) was perfused through the heart, followed by fixation with 4% paraformaldehyde (pH 7.4). The sciatic nerve samples were then extracted and soaked in 4% paraformaldehyde at 4°C overnight for fixation. This was followed by dehydration in a 30% sucrose solution until the samples sank to the bottom. A 30 μm coronal section of the brain sample was performed using a cryomicrotome (NX50, Thermo Fisher, USA) and stored in a -80°C freezer. For immunohistochemical staining, frozen nerve sections were rewarmed for 30 minutes at room temperature and washed 3 times with PBS for 10 minutes each. The nerve films were permeabilized with 0.3% Triton X-100 for 15 min at room temperature, then blocked with 5% goat serum (36119ES10, Yeasen, China) for 2 hours, followed by three additional PBS washes of 10 minutes each. Sections were then incubated overnight at 4°C with neurofilament-L (NF-L) rabbit monoclonal antibodies (1:200, Cat. No. 2837, CST, USA) and myelin basic protein (MBP) rat monoclonal antibodies (1:400, MAB386, Millipore, USA) overnight at 4°C. Following incubation, the sections were washed three times with PBS for 10 minutes each and then incubated with goat anti-rabbit Alex Fluor 488 (1:800, Cat. No. ab150077, Abcam, UK) and goat anti-rat Alex Fluor 594 (1:800, Cat. No. ab150160, Abcam, UK) conjugated fluorescent secondary antibodies for 2 hours at room temperature. After three final PBS washes, sections were mounted using a mounting solution containing 4’,6-diamidino-2-phenylindole (DAPI).

Cells that were dosed for 3 days after axonal degeneration were treated with sterile 4% paraformaldehyde (pH 7.4) for 30 minutes after aspirating the culture medium. The cells were then washed with PBS 3 times for 10 minutes each time. Permeabilize the membrane with 0.1% Triton X-100 for 15 minutes at room temperature, and then block with 5% goat serum for 2 hours. Subsequently, cells were incubated overnight at 4°C with rabbit anti-β-tubulin (1:400, 5568, CST, USA) and washed 3 times with PBS for 10 minutes each, followed by a goat anti-rabbit Alex Fluor 594 (1:800, ab150080, Abcam, UK) conjugated fluorescent secondary antibody for 2 hours at room temperature. Following this, fluorescein Isothiocyanate (FITC)-labeled phalloidin (100 nM, 40735ES75, Yeasen, China) was added and incubated for 30 minutes at room temperature. Finally, after 3 PBS washes, the cells were mounted with mounting solution containing DAPI. Images were observed and captured using a virtual digital slide scanning system (Leica, Germany) and an Olympus camera (Olympus, Japan). The sample size in this experiment (n ≥ 3) was chosen based on previous studies [43, 44].

### 2.9 Cell Mitochondrial Extraction and Western Blot

Mitochondria were extracted from the collected cells using the mitochondrial extraction kit (C3601, Beyotime, China). The extracted mitochondria were lysed in the mitochondrial lysis buffer provided by the kit. Subsequently, the protein content of each sample was quantified using the enhanced bicinchoninic acid (BCA) protein assay kit (P0009, Beyotime, China). Total proteins were separated by 12.5% sodium dodecyl sulfate-polyacrylamide gel electrophoresis (SDS-PAGE) (PG213, Epizyme Biomedical, China) and then transferred to a polyvinylidene difluoride (PVDF) membrane (IPFL00010, Immobilon, USA). The membrane was blocked with 5% non-fat milk at room temperature for 2 hours and then incubated overnight at 4°C with the respective primary antibodies. The following primary antibodies were used: cytochrome c oxidase IV (COX IV) rabbit polyclonal antibody (1:1000, A6564, Abclone, China), histone H3 rabbit monoclonal antibody (1:1000, A17562, Abclone, China), armadillo repeat containing X-linked 1 (ARMCX1) rabbit polyclonal antibody (1:1000, 20193-1-AP, Proteintech, China), fasciculation and elongation protein zeta 1 (FEZ1) rabbit polyclonal antibody (1:1000, HA500531, HuaBio, China), disrupted in schizophrenia 1 (DISC1) rabbit monoclonal antibody (1:1000, A4678, Abclone, China), trafficking kinesin protein 1 (TRAK1) rabbit polyclonal antibody (1:500, 13987-1-AP, Proteintech, China), SNPH rabbit polyclonal antibody (1:2000, A12300, Abclone, China), syntabulin (SYBU) rabbit polyclonal antibody (1:2000, 16972-1-AP, Proteintech, China). After that, the membrane was washed three times with Tris-buffered saline with Tween-20 (TBST) buffer containing 0.1% Tween 20 (each wash for 10 minutes). The membrane was then incubated with horseradish peroxidase (HRP)-conjugated goat anti-rabbit secondary antibody (1:5000, AS014, Abclone, China) for 2 hours. Subsequently, the membrane was washed three times with TBST buffer containing 0.1% Tween 20 (each wash for 10 minutes). According to the instructions of the enhanced chemiluminescence (ECL) chemiluminescence kit (PK10003, Proteintech, China), the developing working solution was prepared, and the PVDF membrane was analyzed. Protein signals were detected using a scanning densitometer (SH-523, SHST, China). Quantitative analysis was performed using ImageJ software. The sample size in this experiment (n ≥ 3) was chosen based on previous studies [45].

### 2.10 Molecular Docking

The three-dimensional structures of strychnine were downloaded from PubChem (https://pubchem.ncbi.nlm.nih.gov/), and the stereo structure of the proteins was downloaded from the PDB database (https://www.rcsb.org/). Then, AutoDock software (4.2.6) was used to complete the docking analysis between the molecular target and the small molecule drug. Finally, PyMOL software (3.0) was used to verify the molecular docking between small drug molecules and protein macromolecules.

### 2.11 Statistical Analysis

The experimental data collected in this study were expressed as results in mean ± standard error of the mean. When data followed a normal distribution, the t-test (for two groups) and one-way analysis of variance with Dunnett's multiple comparison test (for three or more groups) were applied. When the two groups did not meet normality assumptions, the non-parametric Mann-Whitney test was used. The statistical analysis methods used in each figure were clarified in each legend. Sample sizes were not predetermined using statistical methods. Prism (9.0, GraphPad Software, USA) was used for statistical analyses, and Adobe Illustrator (Adobe, USA) was used for graphics. In all analyses, ns indicates no statistical difference, ***P < 0.001, **P < 0.01, and *P < 0.05 indicates a statistical difference.

## 3 RESULT

### 3.1 Strychni Semen and Strychnine Improved Spatial Memory Deficits in Mice after BCAS

**Fig. (1):**
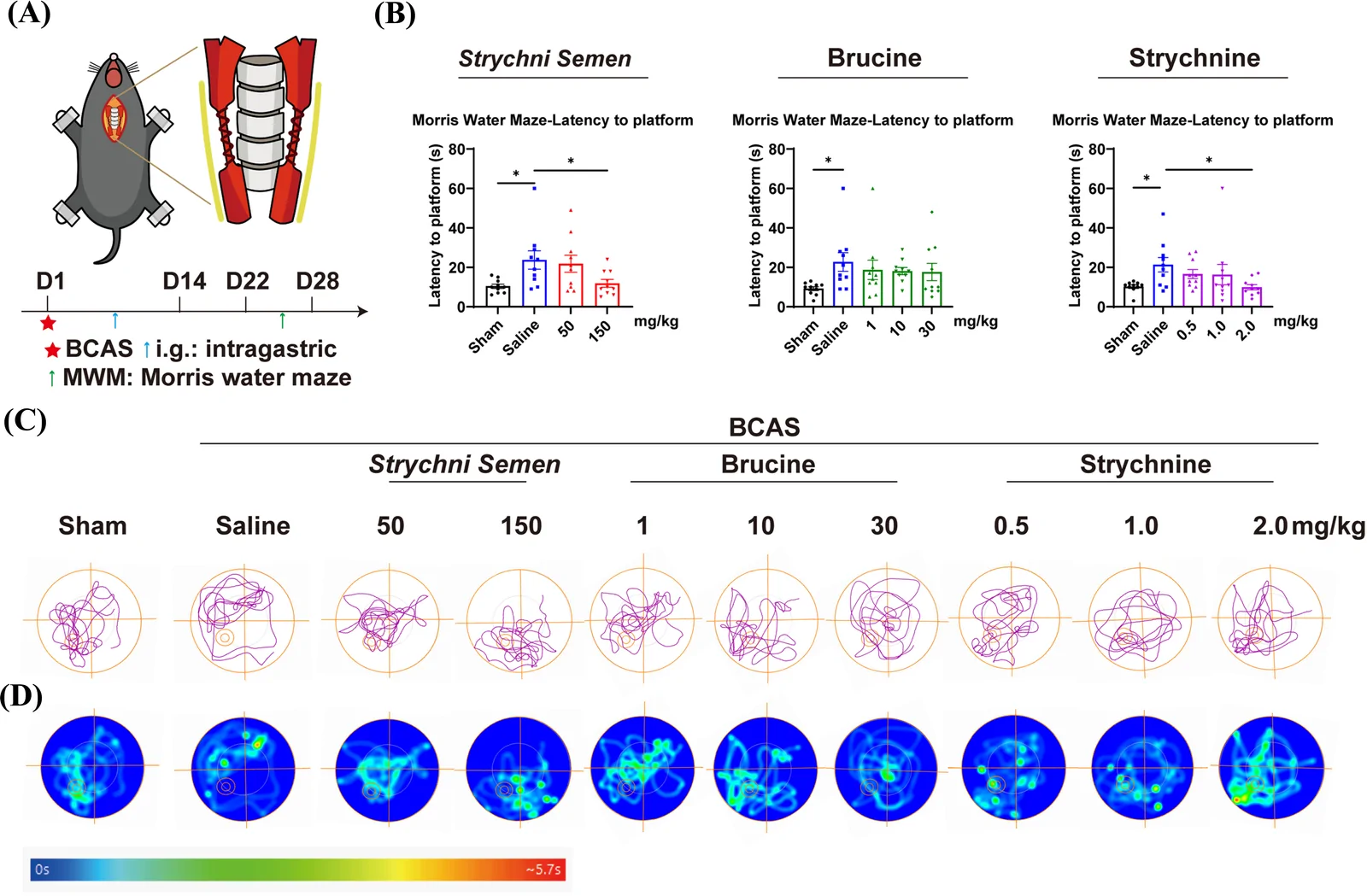
The effects of *Strychni Semen* and its active compounds on the spatial memory ability of mice subjected to BCAS. (**A**) Experimental procedure diagram. (**B**) Latency to reach the target platform in the Morris water maze test (n = 10 mice). (**C**) Representative swimming path diagrams of the mice on the test day. (**D**) Heatmaps of the swimming path on the test day. *P < 0.05. Statistical analysis was performed using one-way analysis of variance with Dunnett’s multiple comparisons test. Error bars represent standard error of the mean. BCAS: bilateral carotid artery stenosis.

To determine the effects of *Strychni Semen* and its active compounds (strychnine and brucine) on spatial memory ability in BCAS mice, the MWM test was conducted (Fig. **1A**). Compared to the sham group, BCAS mice displayed a significantly prolonged average latency to reach the platform, indicating a spatial memory deficit (Fig. **1B**). Administration of *Strychni Semen* by gavage for 14 consecutive days at a dosage of 150 mg/kg, a dosage comparable to clinical usage in humans, resulted in a reduction of the latency to platform in BCAS mice (Figs. **1B**-**D**). This suggests that *Strychni Semen* confers a protective effect against cognitive dysfunction in VaD. Notably, daily gavage administration of strychnine, but not brucine, also improved spatial memory ability in BCAS mice (Figs. **1B**-**D**). These findings indicate that *Strychni Semen* and its active compound, strychnine, can ameliorate spatial memory deficits in BCAS mice, highlighting their potential benefits in managing cognitive impairments associated with VaD.

### 3.2 Strychni Semen and Strychnine Relieved White Matter Injury in BCAS Mice

**Fig. (2):**
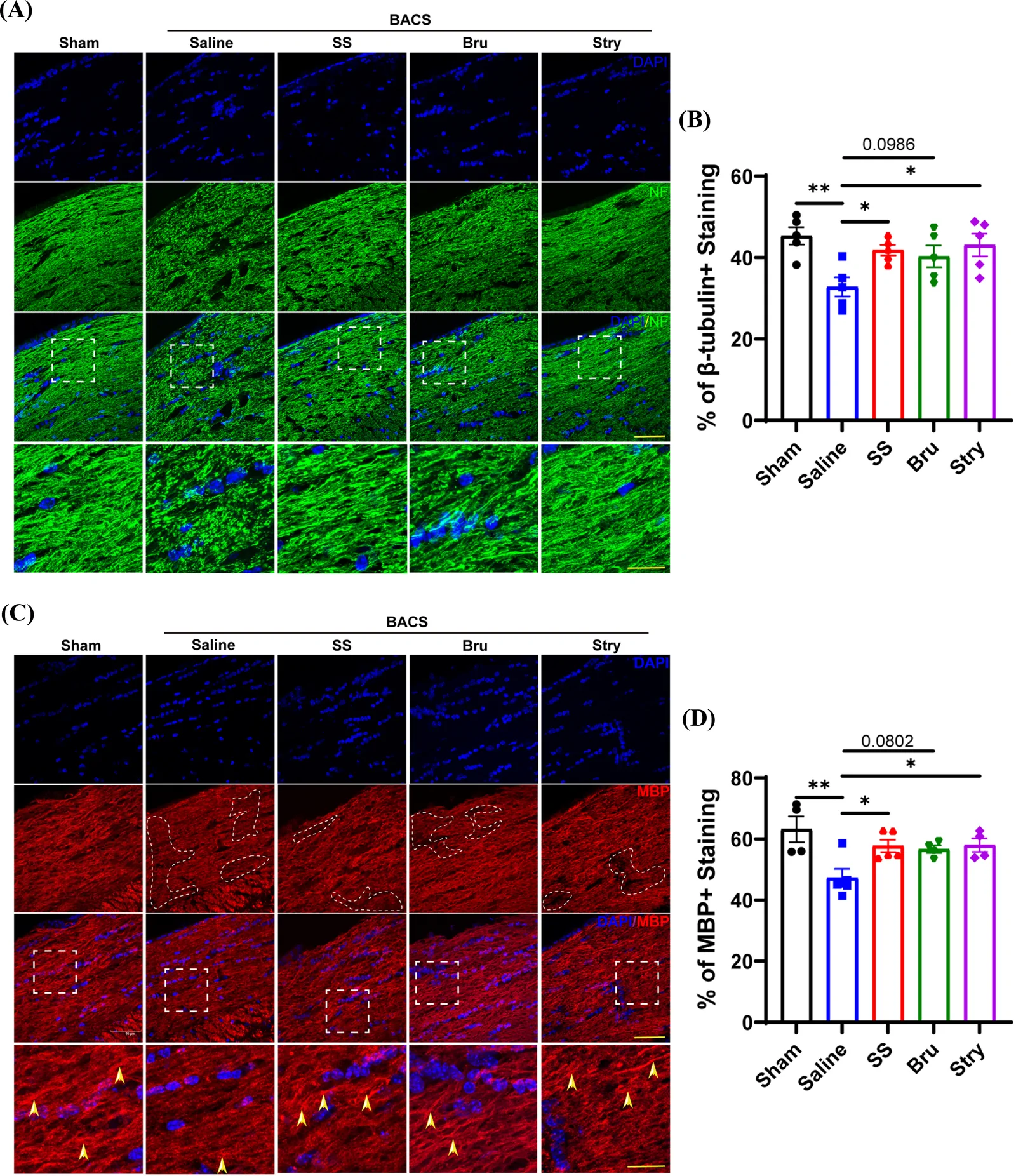
The effects of *Strychni Semen* and its active compounds on axon degeneration and demyelination in mice subjected to BCAS. (**A**) Representative images showing NF expression in the corpus callosum of BCAS mice after administration of *Strychni Semen* and its active compounds. (**B**) The percentage of NF fluorescence area above threshold relative to the total corpus callosum area in different groups (n = 5 mice). (**C**) Representative images showing MBP expression in the corpus callosum of BCAS mice after administration of *Strychni Semen* and its active compounds. (**D**) The percentage of MBP fluorescence area above threshold relative to the total corpus callosum area in different groups (saline group, n = 4 mice; saline group, n = 5 mice; SS group, n = 5 mice; Bru group, n = 4 mice; Stry group, n = 4 mice;). **P < 0.01, *P < 0.05. Scale bar: 50 μm and 20 μm. Statistical analysis was performed using one-way analysis of variance with Dunnett’s multiple comparisons test. Error bars represent standard error of the mean. BCAS: bilateral carotid artery stenosis; Bru: brucine (30 mg/kg); DAPI: 4’,6-diamidino-2-phenylindole; MBP: myelin basic protein; NF: neurofilament; SS: *Strychni Semen* (150 mg/kg); Stry: strychnine (2.0 mg/kg).

Given the pathological significance of white matter injury in VaD, we employed immunostaining of axons and myelin in the corpus callosum to assess the impact of *Strychni Semen* and its active compounds on white matter injury. BCAS mice exhibited a reduction and fragmentation of immunofluorescence signals of the axonal marker NF, indicative of cortical axonal injury (Figs. **2A** and **B**). Treatment with *Strychni Semen* and its active compound, strychnine, reversed the loss of NF immunofluorescence following BCAS, demonstrating a protective effect against axonal injury (Figs. **2A** and **B**). Similarly, BCAS-induced demyelination was observed through the loss and fragmentation of myelin sheaths, as indicated by myelin basic protein (MBP) labeling (Figs. **2C** and **D**). Again, treatment with *Strychni Semen* and strychnine mitigated myelin degeneration after BCAS, as evidenced by the increased MBP immunofluorescence (Figs. **2C** and **D**). Collectively, these findings suggest that *Strychni Semen* and strychnine have the potential to alleviate white matter injury in mice subjected to BCAS, providing a protective effect on both axons and myelin.

### 3.3 Strychni Semen and Strychnine Promoted Axon Regeneration in vivo

**Fig. (3):**
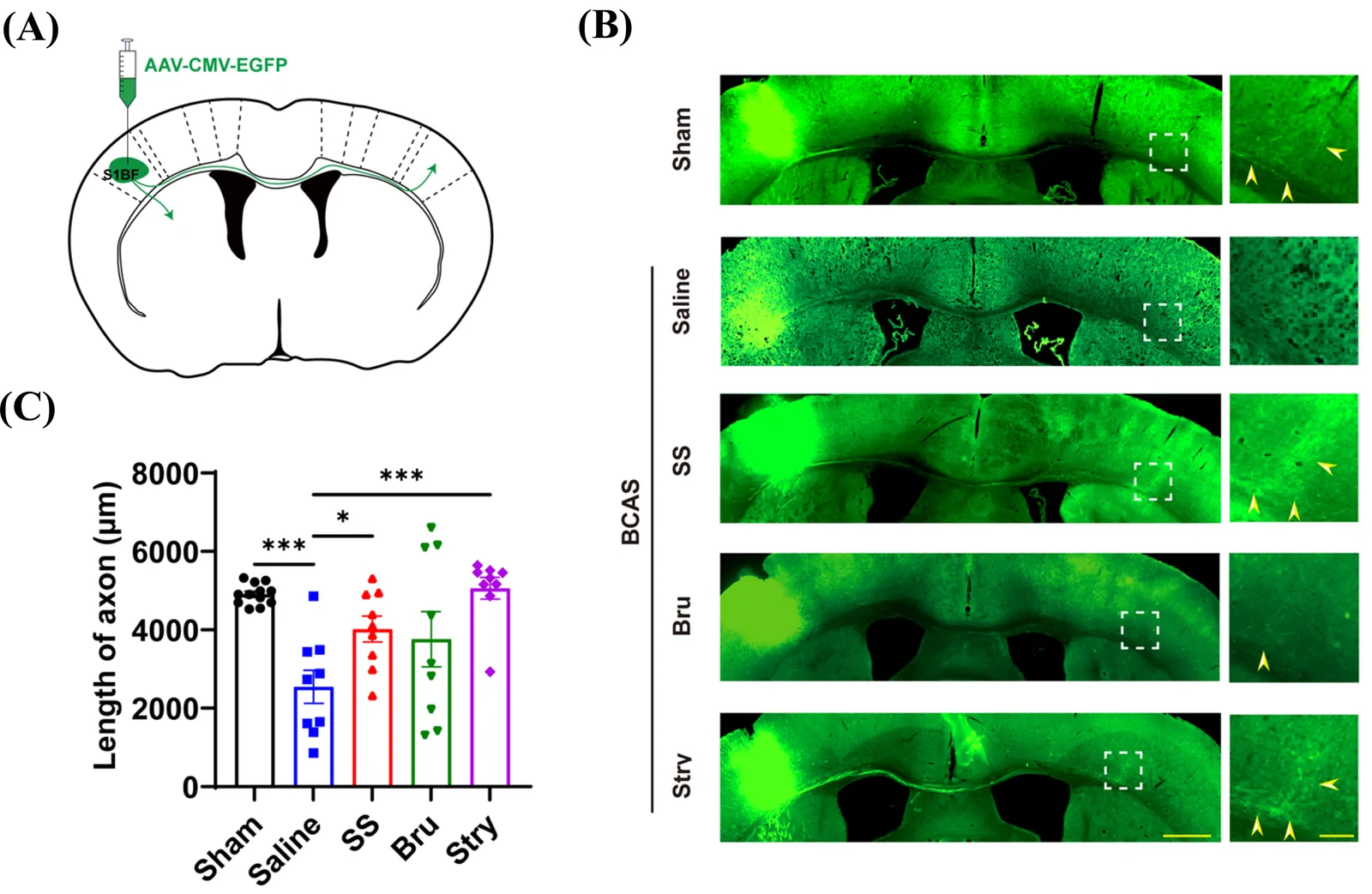
The effects of *Strychni Semen* and its active compounds on the interhemispheric neural connectivity in mice subjected to BCAS. (**A**) Schematic diagram showing virus (AAV-CMV-EGFP) injection in the cortex. (**B**) Representative images showing EGFP expression in the corpus callosum of mice after administration of *Strychni Semen* and its active compounds. (**C**) The length of the longest EGFP-positive axons in different groups (n =3 or 4 mice per group and three different coronal sections from each animal brain are included). ***P < 0.001, *P < 0.05. Scale bar: 500 μm and 100 μm. Statistical analysis was performed using one-way analysis of variance with Dunnett’s multiple comparisons test. Error bars represent standard error of the mean. BCAS: bilateral carotid artery stenosis; Bru: brucine (30 mg/kg); SS: *Strychni Semen* (150 mg/kg); Stry: strychnine (2.0 mg/kg).

Given the limited strategies available for promoting axonal regeneration in the brain, we focused our subsequent research on the effect of *Strychni Semen* on axonal regeneration. To further investigate this effect, we examined cortical neuronal connections to the contralateral hemisphere in mice subjected to the BCAS model [46]. One day before inducing BCAS, enhanced green fluorescent protein (EGFP)-expressing viruses were injected into the primary somatosensory barrel cortex (S1BF) region of the mice (Fig. **3A**). Four weeks later, coronal brain sections of these mice were prepared for analysis. In the contralateral hemisphere of the sham group mice, fibrous EGFP fluorescent signals were clearly observed (Figs. **3B** and **C**). In contrast, the BCAS group exhibited a significant reduction of EGFP fluorescent signals in the contralateral hemisphere, indicating impaired interhemispheric neural connections after BCAS (Figs. **3B** and **C**). Notably, *Strychni Semen* and strychnine rescued neuronal connectivity between hemispheres in BCAS mice (Figs. **3B** and **C**). Conversely, brucine did not provide a protective effect against the deficiency in neuronal connectivity. These results indicate that *Strychni Semen* and strychnine promote axonal regeneration *in vivo*, offering potential therapeutic benefits for the recovery of interhemispheric neural connections in VaD.

### 3.4 Strychnine Promoted Axon Regeneration in vitro

**Fig. (4):**
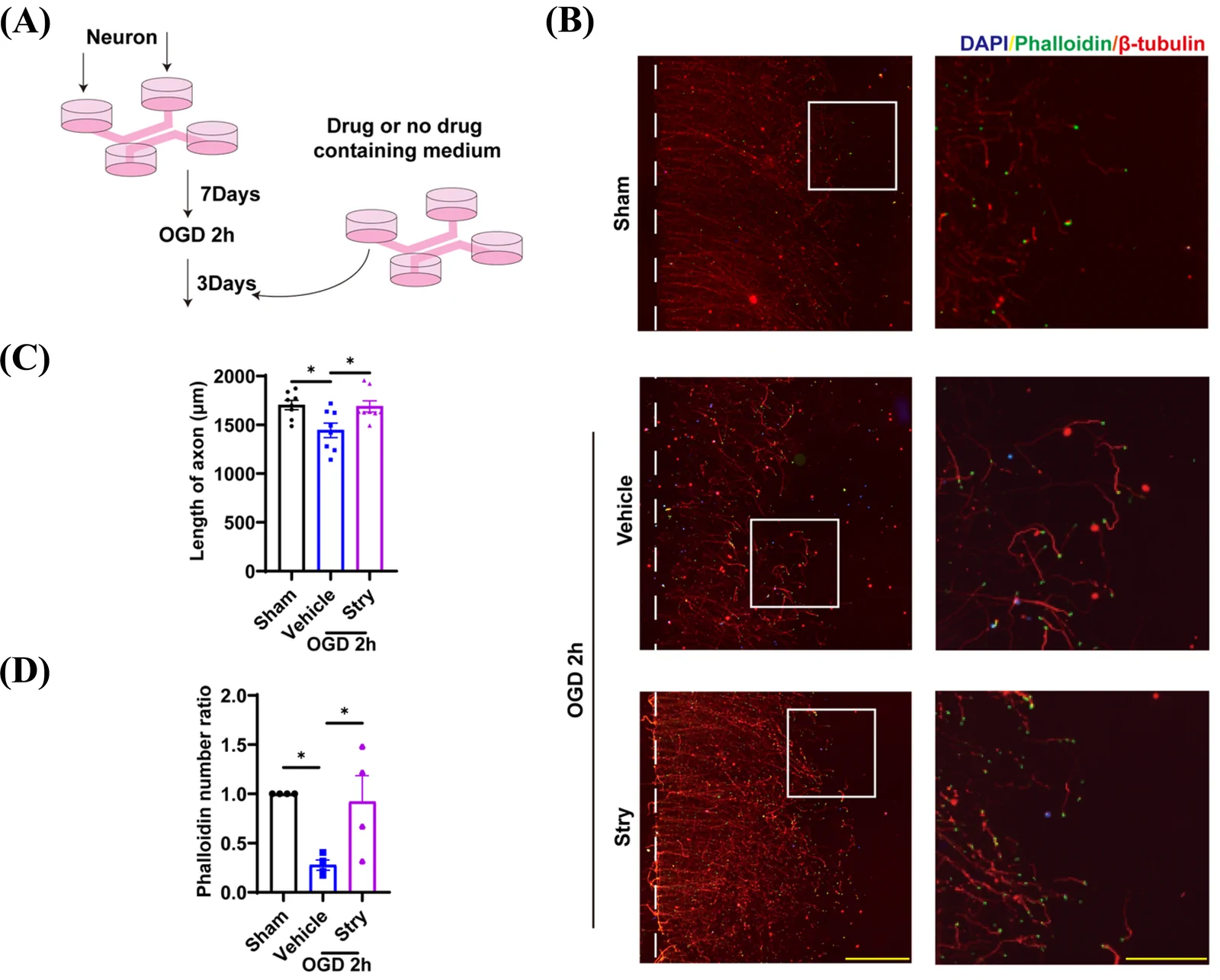
Strychnine promoted axon regeneration in cultured cortical neurons after OGD. (**A**) Schematic diagram showing the experimental setup. (**B**) Representative immunofluorescence images of axons. (**C**) The length of the longest axons in different groups (n = 8 independent experiments, each value presents the average length of the 10 longest axons in one experiment). (**D**) The percentage of phalloidins-positive axons in all axons (n = 4 independent experiments). *P < 0.05. Scale bar: 500 μm and 200 μm. Statistical analysis was performed using one-way analysis of variance with Dunnett’s multiple comparisons test. Error bars represent standard error of the mean. DAPI: 4’,6-diamidino-2-phenylindole; OGD: oxygen-glucose deprivation; Stry: strychnine (10 μM).

 To further evaluate the impact of strychnine on axonal regeneration, we cultured primary cortical neurons *in vitro* and established an OGD model to simulate VaD-induced axon degeneration (Fig. **4A**). Compared with the control group, OGD significantly reduced axonal length (as measured by β-tubulin III and FITC-phalloidin labeling; Figs. **4B** and **C**) and decreased the number of FITC-phalloidin-labeled growth cones (Figs. **4B** and **D**). These observations indicate axonal degeneration following OGD. Notably, strychnine not only elongated the axonal length (Figs. **4B** and **C**) but also increased the number of growth cones (Figs. **4B** and **D**) after OGD. These results support the axon regeneration-promoting effect of strychnine under conditions mimicking VaD-induced neuronal damage.

### 3.5 Strychnine Enhanced Axonal Mitochondrial Motility after OGD

**Fig. (5):**
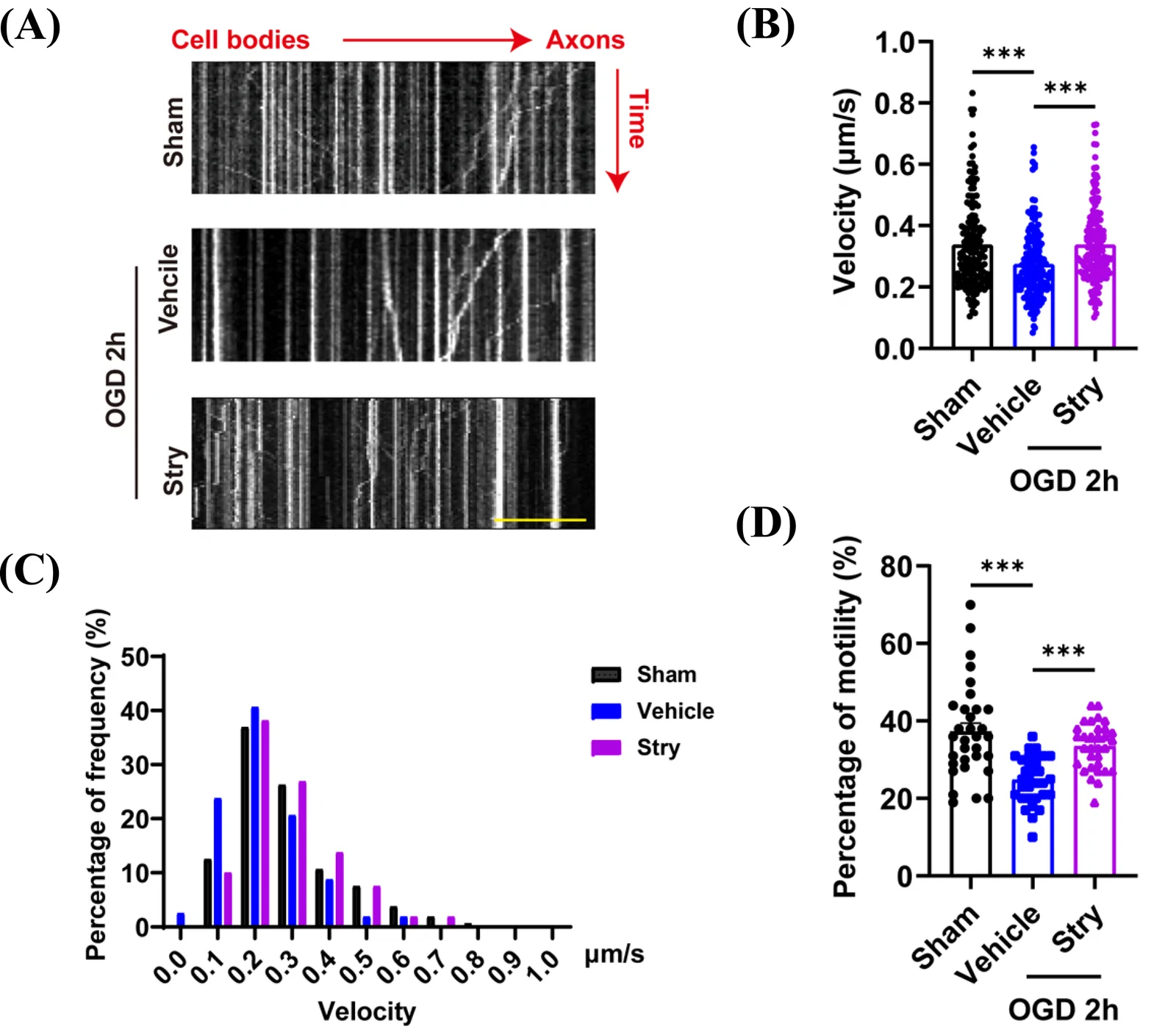
Strychnine promoted axonal mitochondrial transport in cultured cortical neurons after OGD. (**A**) Representative images showing mitochondrial movement trajectories in axons. Scale bar: 50 μm. (**B**) Mitochondrial movement velocity in axons. (n = 160 mitochondria from 4 independent experiments for each group). (**C**) Velocity distribution histogram showing the percentage of mitochondria across velocity bins (**D**) The percentage of mobile mitochondria in axons. (n = 40 axons from 4 independent experiments for each group). ***P < 0.001. Statistical analysis was performed using one-way analysis of variance with Dunnett’s multiple comparisons test. Error bars represent standard error of the mean. DAPI: 4’,6-diamidino-2-phenylindole; OGD: oxygen-glucose deprivation; Stry: strychnine (10 μM).

Given the critical role of mitochondrial transport in axonal regeneration [47], we further evaluated the effect of strychnine on axonal mitochondrial transport. We quantified both the velocity and percentage of moving mitochondria in the axons of cultured cortical neurons. Compared to the control group, OGD significantly reduced mitochondrial motility in axons, reflected by decreased mitochondrial velocity and a lower percentage of mobile mitochondria (Fig. **5**). Notably, treatment with strychnine markedly rescued this deficit. Specifically, strychnine increased mitochondrial velocity (Figs. **5A**, **B**, and **C**) and enhanced the percentage of moving mitochondria (Figs. **5A** and **D**), effectively rescuing the impaired mitochondrial transport caused by OGD.

### 3.6 Strychnine Appears to Promote Axon Regeneration by Enhancing Axonal Mitochondrial Motility after OGD

**Fig. (6):**
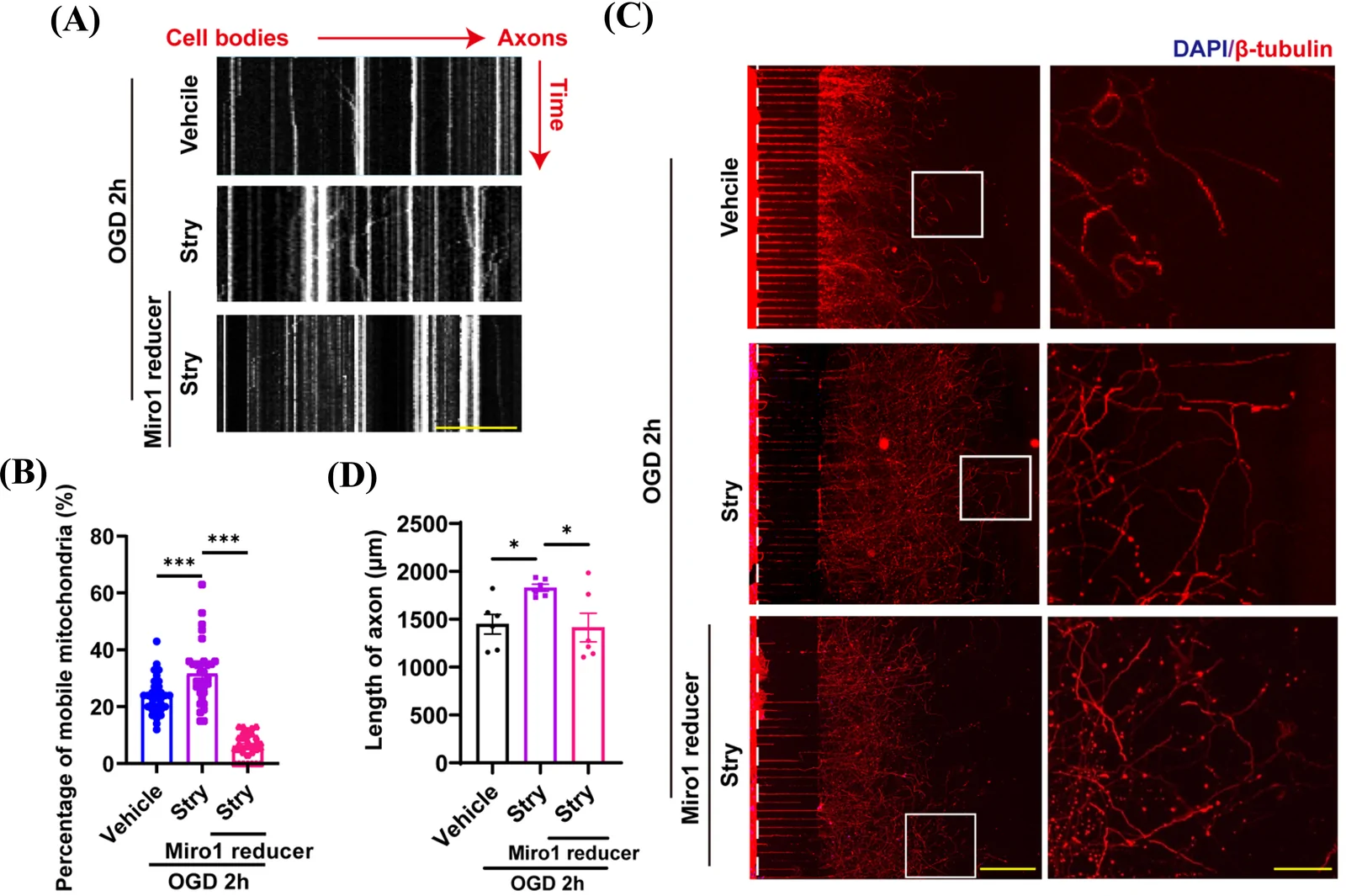
Inhibiting mitochondrial movement by Miro1 reducer abolished the axon regeneration-promoting effect of strychnine after OGD. (**A**) Representative images showing mitochondrial movement trajectories in axons. (**B**) The percentage of mobile mitochondria in axons. (n = 40 axons from 4 independent experiments for each group). (**C**) Representative immunofluorescence images of axons. Scale bar: 250 μm. (**D**) The length of the longest axons in different groups (n = 6 independent experiments, each value presents the average length of the 10 longest axons in one experiment). ***P < 0.001, *P < 0.05. Scale bar: 400 μm and 100 μm. Statistical analysis was performed using one-way analysis of variance with Dunnett’s multiple comparisons test. Error bars represent standard error of the mean. DAPI: 4’,6-diamidino-2-phenylindole; Miro1: ras homolog family member T1; OGD: oxygen-glucose deprivation; Stry: strychnine (10 μM).

To verify the contribution of mitochondrial transport to the axon regeneration-promoting effect conferred by strychnine, we inhibited axonal mitochondrial movement by using Miro1 reducer. Miro1 reducers is a small molecular compound that can specifically bind to Miro1 proteins, which a key protein that anchors mitochondria to microtubules and facilitates their transport along the cytoskeleton [48, 49]. The binding of Miro1 reducers induced the proteasomal degermation of Miro1, thereby inhibiting axonal mitochondrial movement [48, 50]. As expected, Miro1 reducer suppressed the enhanced mitochondrial movement by strychnine (Figs. **6A** and **B**) and abolished its axon regeneration-promoting effect (Figs. **6C** and **D**). These findings underscore the role of strychnine in promoting axonal regeneration, which may occur partially through enhanced axonal mitochondrial motility.

**Fig. (7):**
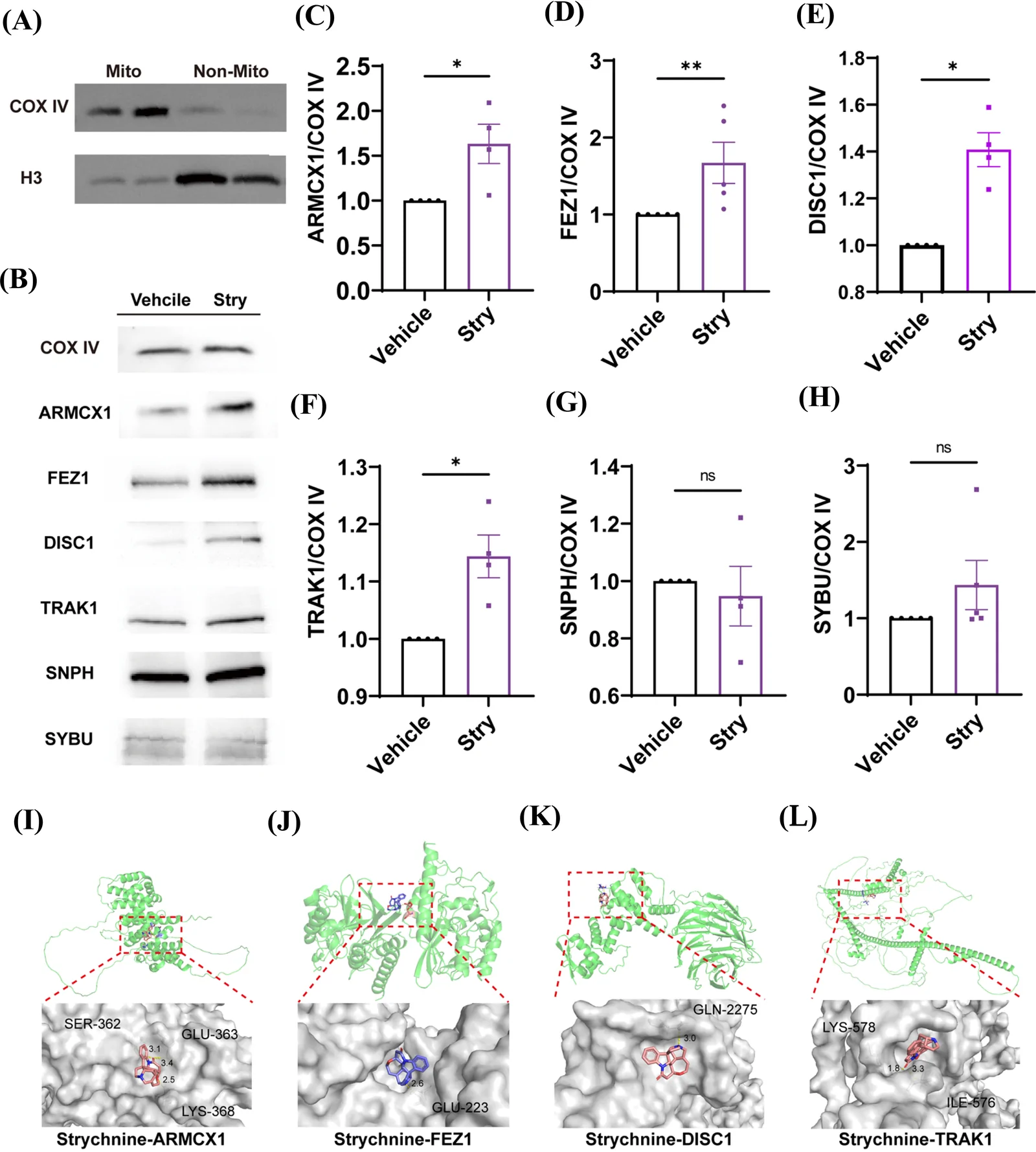
Strychnine recruited transport-related proteins to mitochondria in cultured cortical neurons after OGD. **A**: Representative blots showing mitochondrial isolation from cells. **B**: Representative blots showing protein expressions in mitochondria. **C**-**H**: Quantification of protein expression level in mitochondria ARMCX1 (n = 4 independent experiments) (**C**); FEZ1 (n = 5 independent experiments) (**D**); DISC1 (n = 4 independent experiments) (**E**); TRAK1 (n = 4 independent experiments) (**F**); SNPH (n = 4 independent experiments) (**G**); SYBU (n = 5 independent experiments) (**H**). I-L: Molecular docking analysis of strychnine with ARMCX1(**I**), FEZ1(**J**), DISC1(**K**), TRAK1(**L**). ns indicates no statistical difference, **P < 0.01, *P < 0.05. Statistical analysis was performed using Mann-Whiteny test. Error bars represent standard error of the mean. ARMCX1: armadillo repeat containing X-linked 1; COX IV: cytochrome c oxidase IV; DISC1: disrupted in schizophrenia 1; FEZ1: fasciculation and elongation protein zeta 1; GLN-2275: glutamine residue at 2275; GLU-223: glutamicacid residue at 223; GLU-363: glutamicacid residue at 363; ILE:576: isoleucine residue at 576; LYS-368: lysine residue at 368; LYS-578: lysine residue at 578; OGD: oxygen-glucose deprivation; SER-362: serine residue at 362; SNPH: syntaphilin; Stry: strychnine (10 μM); SYBU: syntabulin; TRAK1: trafficking kinesin protein 1.

To further investigate potential molecular machinery involved in strychnine-rescued axonal mitochondrial transport, we isolated mitochondria from cultured cortical neurons and examined the expression levels of several mitochondrial transport-related proteins [51-53] in mitochondria (Fig. **7A**). Following OGD, western blot analysis revealed that strychnine treatment promoted the translocation of ADMAX1, FEZ1, DISC1, and TRAK1 to mitochondria (Figs. **7B**-**H**). Furthermore, we used the molecular docking technique to calculate the binding affinity of these proteins with strychnine separately. The results showed that strychnine potentially binds to these proteins with different affinity (ARMCX1: -4.66 kcal/mol; FEZ1: -6.23 kcal/mol; DISC1: -6.52 kcal/mol; TRAK1: -5.18 kcal/mol) (Figs. **7I**-**L**). Specifically, strychnine forms one hydrogen bond with glutamate residue at 223 position of FEZ1(Fig. **7J**) and one hydrogen bond with glutamine residue at 2275 position of DISC1 (Fig. **7K**). These findings indicate that strychnine can significantly promote the translocation of various transport motor proteins to mitochondria, reinforcing its role in enhancing mitochondrial transport. Notably, the proteins FEZ1 and DISC1, in particular, seem to play more central roles in this process, as evidenced by their stronger binding affinities and specific interactions with strychnine.

## 4 DISCUSSION

VaD, characterized by cognitive decline and white matter injury, represents a significant challenge in the field of neurological health [54, 55]. *Strychni Semen* has been shown to have therapeutic effects in ischemic stroke [56]; however, the impact of *Strychni Semen* on cognitive function after chronic hypoperfusion was not fully understood. Here, we found that *Strychni Semen* and its active compound, strychnine, improved the spatial memory of mice subjected to BCAS (Fig. **1**). Consistently, *Strychni Semen* and strychnine rescued the loss of axon (labeled by NF) and myelin (labeled by MBP) within the corpus callosum following BCAS, indicating their protective effect against white matter injury in VaD (Fig. **2**). Interestingly, strychnine, but not brucine, recapitulated the neuroprotective effect of *Strychni Semen* in mice subjected to BCAS. Compared to brucine, strychnine has two additional methoxy groups in its structure, which enhance the drug's lipophilicity and its ability to cross the blood-brain barrier [57, 58]. The structural distinctions between strychnine and brucine may account for their differing efficacies in the treatment of VaD. Although brucine has been reported to have antioxidant and anti-apoptotic effects [59-62], these actions may not be critical to the recovery from white matter injury [63-65]. Taken together, our results shed light on a promising future application fo*r Strychni Semen* and strychnine in the treatment of VaD.

Although accumulating evidence supports the neuroprotective effect of *Strychni Semen* [66, 67], the mechanisms behind these effects were not fully understood. This study provides the first evidence that *Strychni Semen* and strychnine can promote axonal regeneration in central neurons both *in vivo* and *in vitro* (Figs. **3** and **4**). Along with axon repair, *Strychni Semen* and strychnine also increased MBP expression in the corpus callous. This effect may be indirectly stemmed from axon regeneration since regenerated axons can release bio-factors activating remyelination [68, 69]. The direct impact of *Strychni Semen* and strychnine on oligodendrocytes and myelination needs further investigation. In the context of axotomy models, the overexpression of growth-associated proteins such as growth-associated protein 43 (GAP-43) and its homolog GAP-23 has been shown to rejuvenate mature central neurons, restoring their capacity for axonal regrowth [70, 71]. Studies using a mouse model of optic nerve crush injury have demonstrated that manipulating the PTEN/mTOR pathway through virus-assisted *in vivo* gene knockout can effectively promote axon regeneration within the CNS [72, 73]. Moreover, recent research has highlighted the therapeutic potential of mitochondrial transplantation, stem cell therapy, and neural progenitor cell transplantation in spinal cord injury models, underscoring their efficacy in fostering axonal regeneration [74-76]. Despite these advancements, current strategies for enhancing central axonal regeneration predominantly revolve around genetic manipulation and surgical interventions, with small-molecule drugs playing a less prominent role [77, 78]. Gene therapy and stem cell therapy carry potential risks and limitations. Gene therapy may provoke immune reactions from viral vectors, cause insertional mutagenesis, face challenges in precise gene targeting, and raise ethical concerns over heritable edits [79, 80]. Stem cell therapies risk tumor formation from undifferentiated cells, immune rejection, incomplete or abnormal cell differentiation, and ethical debates around embryonic sources [81, 82]. Both therapies are hindered by high costs, technical complexity in manufacturing, limited long-term safety data, and regulatory barriers. Since *Strychni Semen* is clinically used, this work prioritizes its therapeutic potential in VaD and post-stroke management, opening new avenues for developing non-invasive treatment options aimed at promoting recovery from CNS injuries.

Notably, our research demonstrates that strychnine can promote mitochondrial movement within axons. These findings provide new experimental evidence to support the clinical application of *Strychni Semen* in treating cerebral ischemia-related disorders. The pathological hallmarks of VaD encompass neuroinflammatory responses, neuronal apoptosis, and synaptic reorganization [83, 84]. In light of the exceptionally high energy demands of the neuron [85], our research has prioritized elucidating the therapeutic potential of mitochondrial modulation in VaD intervention. Previous studies have shown that enhancing mitochondrial mobility in axons through genetic manipulation can boost axonal regeneration [86]. Our findings showed that strychnine not only promotes axonal mitochondrial movement (Fig. **5**) but also enhances the translocation of proteins associated with mitochondrial transport (Fig. **7**). Importantly, inhibiting axonal mitochondrial movement nullifies the axonal regeneration-promoting effects of strychnine following OGD (Fig. **6**). These results indicate a potential role of strychnine in supporting axonal regeneration through enhanced axonal mitochondrial transport. Molecular docking studies underscore the critical roles of DISC1 and FEZ1 in these processes. Specifically, FEZ1 acts as an adaptor for the motor protein Kinesin-1 [87], actively participating in mitochondrial transport within axons [88]. Therefore, enhancing the translocation of FEZ1 to mitochondria significantly promotes axonal mitochondrial mobility. Moreover, DISC1 interacts with FEZ1 to regulate mitochondrial transport [89], implying a synergistic effect between these two proteins in promoting axonal regeneration. Previous reports have highlighted the protective role of FEZ1 against post-stroke depression [90] and the importance of DISC1 in traumatic brain injury [91]. Our results further underscore their involvement in post-stroke cognitive dysfunction and VaD, indicating that these proteins may serve as potential therapeutic targets for neurodegenerative conditions. Collectively, our study provides novel insights into the mechanisms by which strychnine promotes axonal regeneration, highlighting the critical role of mitochondrial transport in this process.

In our study, we identified several limitations that warrant further exploration. Firstly, the assessment of *Strychni Semen* and its active compounds in VaD was limited to only 2-3 dosages. To better inform future clinical applications, a more comprehensive dose-response curve necessitates testing across additional dosage points. It's important to note that our current findings do not completely rule out the possibility that brucine may help strychnine in exerting its effects. Future research will be necessary to compare the outcomes of using strychnine alone *versus* in combination with brucine to determine if there is indeed an additive or synergistic effect when both compounds are used together. Our evaluation of the mechanisms was confined to *in vitro* settings. Future investigations should utilize Mito-GFP transgenic mice with *in vivo* two-photon imaging techniques to validate the impacts of *Strychni Semen* on mitochondrial dynamics post-VaD. Moreover, while our molecular docking analysis provided preliminary insights into the interactions between strychnine and FEZ1/DISC1, these findings remain predictive. Future research employing neuron-specific epitope-tagged FEZ1/DISC1 constructs, such as GFP fusion proteins, coupled with live-cell imaging, would offer more direct evidence for elucidating their dynamic subcellular localization. Additionally, to pinpoint the direct targets of strychnine, future studies must integrate Co-IP with affinity proteomics. Based on an enhanced understanding of the underlying molecular mechanisms, genetic manipulations aimed at blocking mitochondrial transport *in vivo* are needed to establish a causal relationship between mitochondrial transport, axonal regeneration, and cognitive recovery following VaD.

## CONCLUSION

Our findings suggest that *Strychni Semen* improved spatial memory and promoted axonal regeneration in mice subjected to BCAS. Its active component compound strychnine, but not brucine, recapitulated the neuroprotective effect of *Strychni Semen* in mice subjected to BCAS. Strychnine promoted axon regeneration and enhanced mitochondrial transport in primary cultured neurons subjected to OGD. Inhibiting mitochondrial transport reversed the neuroprotective effect of strychnine *in vitro*. These findings collectively suggest that *Strychni Semen* improves spatial memory and promotes axon regeneration following VaD, and that enhanced mitochondrial transport might be involved in its neuroprotective effect against VaD.

## Data Availability

The authors confirm that the data supporting the findings of this research are available within the article.

## LIST OF ABBREVIATIONS

BCAS: Bilateral Carotid Artery Stenosis
CNS: Central Nervous System
MBP: Myelin Basic Protein
MCAO: Middle Cerebral Artery Occlusion
MWM: Morris Water Maze
OGD: Oxygen-glucose Deprivation
TCM: Traditional Chinese Medicine
VaD: Vascular Dementia
